# Gender differences in the impact of anatomical factors on non-contact anterior cruciate ligament injuries: a magnetic resonance study

**DOI:** 10.1186/s12891-024-07390-5

**Published:** 2024-04-04

**Authors:** Xixin Jin, Xujin Wu, Wei Xu, Chang She, Liubing Li, Yongtao Mao

**Affiliations:** Department of Orthopedics, The Second Affiliated Hospital, Soochow University, 1055 Sanxiang Road, Suzhou, Jiangsu 215004 P.R. China

**Keywords:** ACL injury, Tibiofemoral anatomy, MRI, Risk factors, Gender differences

## Abstract

**Purpose:**

To identify MRI-detected anatomical risk factors for non-contact anterior cruciate ligament (ACL) injuries across genders.

**Methods:**

A retrospective analysis was performed on 141 ACL-reconstructed patients (35 females, 106 males) and 142 controls (37 females, 105 males) from January 2020 to April 2022. Inclusion criteria were primary non-contact ACL injuries. The tibial plateau slope, lateral femoral condyle index, Insall-Salvati index, and patellar tendon angle were measured, using binary logistic regression for gender-specific risk evaluation.

**Results:**

Increased lateral tibial plateau slope, reduced intercondylar notch width index, lateral femoral condyle index, and patellar tendon angle correlated with ACL injuries in both genders. The Insall-Salvati index was a significant risk factor in females but not in males.

**Conclusion:**

This study identifies the lateral tibial plateau slope, notch width index, lateral femoral condyle index, and patellar tendon angle at near-extension as risk factors for ACL injuries in both genders, with the Insall-Salvati index also implicated in females.

## Introduction

Anterior cruciate ligament (ACL) injuries are frequently encountered in sports activities [1, 2], with non-contact ACL injuries—occurring without any collision with another person or object—making up 95% of ACL injuries in ball sports [3]. Such injuries are notably common among young athletes, with prevalence rates as high as 3.7% in certain athletic cohorts [4], and female athletes face higher risks, including hormone levels, muscle strength, and anatomical factors [4]. ACL ruptures lead to instability in the knee, and despite reconstructive efforts, the complete prevention of osteoarthritis remains elusive [5]. Consequently, there is a significant interest in exploring the risk factors for ACL injuries to better identify those at risk and enhance prevention efforts.

Studies have been conducted on factors associated with ACL injuries, such as the link between ACL injuries and the tibial plateau slope (TPS), but these studies often present divergent outcomes [6, 7]. Similarly, debates exist regarding whether the shape of the lateral femoral condyle plays a role in ACL injuries [8, 9]. Additionally, the ACL is subjected to increased shearing forces under the patellar tendon when the knee is extended [10, 11], highlighting the importance of examining the relationship between the patellar tendon angle and the patellar index (Insall-Salvati index) in the context of ACL injuries. Moreover, while many anatomical studies overlook gender differences, a handful suggest that the lateral tibial plateau slope (LTPS) correlates with ACL injuries in females but not in males [12], indicating a potential oversight in studies that do not differentiate by gender due to anatomical variances. Thus, this study aims to elucidate the relationship between gender-specific anatomical characteristics and non-contact ACL injuries. Recognizing the challenges in altering gender and anatomical factors, understanding their associated risks can contribute to targeted screening and preventative strategies for athletes, as well as aid in preventing reinjury after ACL reconstruction. Therefore, this research employs magnetic resonance imaging (MRI) to investigate the anatomical risk factors for non-contact ACL injuries across different genders, with a focus on the tibiofemoral joint and patella.

## Methods

A retrospective study was performed on consecutive non-contact ACL reconstruction patients at our institution from January 2020 to April 2022. Inclusion criteria were primary non-contact ACL injuries, focusing on injuries typically due to overexertion or poor sports techniques [13, 14]. Exclusion criteria included: contact ACL injuries, other ligament injuries, skeletal dysplasia, knee fractures, osteoarthritis, prior knee surgeries, incomplete imaging data, and serious systemic diseases. The control group comprised patients without knee injuries, matched by age and gender, who underwent lower limb MRI due to discomfort around the knee joint but had no abnormalities on imaging.

MRI scans were performed using a 1.5 T scanner and a dedicated knee joint coil array (with the scan parameters set as follows: axial T1 phase with a repetition time of 4400ms and echo time of 30ms, sagittal T1 phase with a repetition time of 420ms and echo time of 13ms, and sagittal T2 phase with a repetition time of 3600ms and echo time of 100ms). Slices were set at a thickness of 3 mm, covering the entire anatomical structure between the proximal patella and the distal tibial tuberosity.

**Fig. 1:**
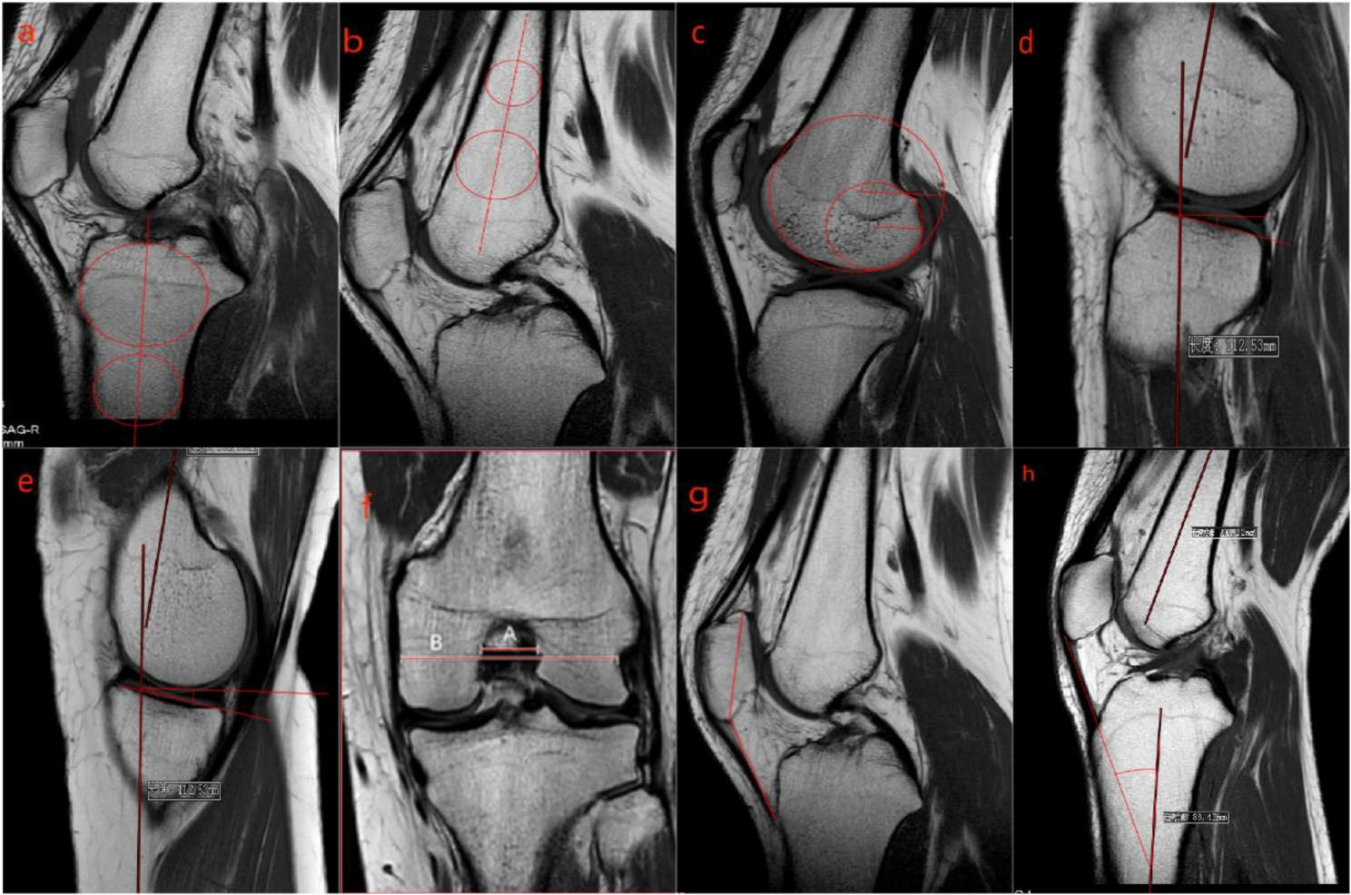
**a**: Determination of the tibial longitudinal axis; **b**: Determination of the femoral longitudinal axis; **c**: Calculation of the LFCI; **d**:LTPS; **e**:MTPS; **f**: NWI; **g**: Insall-Salvati index; **h**: Patellar tendon angle

Anatomical parameters were measured based on previous studies [15], summarized as follows: Two circles were drawn on the tibia, one tangent to the anterior, posterior, and proximal edges of the tibia, and the other tangent to the anterior and posterior edges. The line connecting the centers of the two circles was defined as the longitudinal axis of the tibia, and similarly, the longitudinal axis of the femur was determined. The radii of the lateral condylar flexion circle (LCFCR) and the lateral condylar extension circle (LCECR) were determined based on the curvature of the lateral femoral condyle during flexion and extension, approximated to two circles, to estimate the shape of the lateral femoral condyle. The lateral femoral condyle index (LFCI) was measured by dividing the radius of the flexion circle by the radius of the extension circle, as described by Hodel, S et al. [16]. Measurements of the medial slopes of the tibial plateau (MTPS) and lateral slopes of the tibial plateau were conducted. The notch width index (NWI) was calculated as the ratio of the notch width to the width across both condyles. The Insall-Salvati index was measured as the length of the patellar tendon over the length of the patella, and the patellar tendon angle was defined as the angle between the patella and the tibia shaft. The measurement methods are illustrated in Fig. 1.

### Ethics approval

This study was approved by the Ethics Committee of the Second Affiliated Hospital of Suzhou University, with the ethical review number: JD-HG-2023-41. As this study is an anonymous retrospective study and data analysis, it is not subject to the restrictions of patient informed consent.

### Statistical methods

Data analysis employed regression modeling, using binary logistic models for males and females to evaluate the association between ACL injuries and anatomical factors, with ACL status (0 = normal; 1 = injured) as the dependent variable. Receiver operating characteristic (ROC) curve analysis assessed sensitivity and specificity, identifying optimal cutoff values. Analyses were conducted with SPSS 26.0, considering *P* < 0.05 as statistically significant.

## Results

### Patient selection

**Fig. 2:**
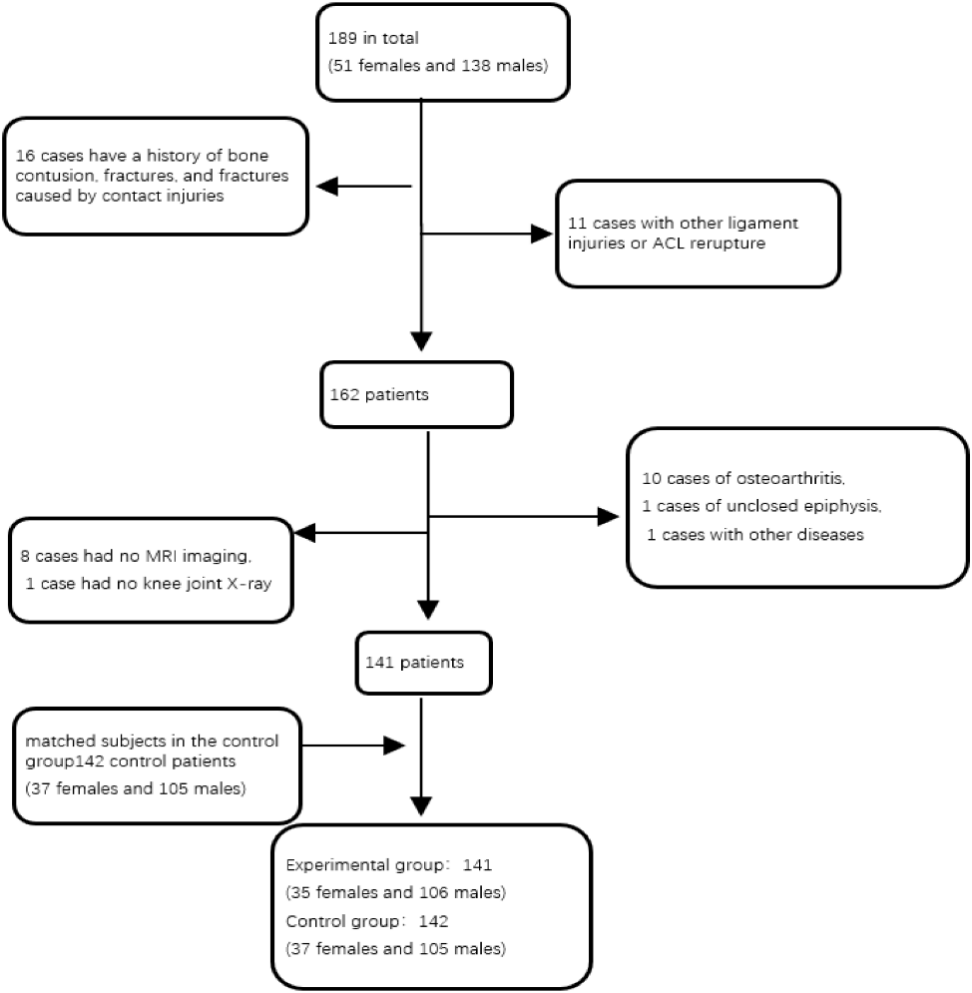
Flowchart of patient selection for this study

Of 189 patients undergoing primary ACL reconstruction, 48 were excluded due to: additional ligament injuries or subsequent ACL rupture (11), contact injuries or fracture history (16), osteoarthritis (10), incomplete MRI data (8), open growth plates (1), missing knee x-rays (1), and ankylosing spondylitis (1). This resulted in 141 patients with ACL injuries (35 females, 106 males) and 142 controls (37 females, 105 males) included. An independent samples T-test showed no significant age difference between groups (31.9 ± 10.1 vs. 33.4 ± 9.9 years, *P* = 0.268) (Fig. 2).

### Binary logistic analysis

The LTPS significantly correlated with ACL injuries in both genders (males *P* < 0.001, females *P* = 0.022). Similarly, the NWI was significantly associated with ACL injuries (females *P* = 0.032, males *P* < 0.001), as was the LFCI (males *P* < 0.001, females *P* = 0.008). The Insall-Salvati index demonstrated significance in females (*P* = 0.001) but not in males (*P* = 0.601) (Table 1).

**Table 1:** Results of logistic analysis for patients

	AVG		P		SE		OR		95%CI	
	M	F	M	F	M	F	M	F	M	F
MTPS	5.667	6.713	0.087	0.533	0.059	0.130	0.903	0.922	0.804–1.015	0.714–1.190
LTPS	6.094	6.428	0.000	0.022	0.058	0.106	1.293	1.275	1.153–1.450	1.035–1.569
NWI	0.275	0.278	0.000	0.032	0.065	0.100	0.767	0.807	0.675–0.872	0.663–0.982
Insall-Salvati	1.067	1.128	0.601	0.001	1.110	0.025	0.560	1.087	0.064–4.929	1.036–1.142
LFCI	0.559	0.557	0.000	0.008	0.036	0.082	1.147	1.244	1.070–1.230	1.058–1.462

(AVG: average; F: Females; M:males; SE: Standard Error)

Considering the patellar tendon angle’s dependence on the knee’s flexion-extension angle [11], we could only measure the patellar tendon angle at the knee flexion-extension angle during MRI imaging. The knee joint angle during imaging is generally in a near-extended position as standard, and patients were divided into two groups based on the knee flexion-extension angle: 0–20° and above 20°. The 0–20° group was taken as the standard for binary logistic regression analysis. The results for both men and women were consistent, showing that the angle of the patellar tendon when the knee flexion-extension angle was 0–20° had significant implications for ACL injuries (males *P* = 0.009, females *P* = 0.013) (Table 2).

**Table 2:** Results of the Patellar tendon angle

		Number of people	P	SE	OR	95%CI
M	Patellar tendon angle at 0–20 ° knee joint	196	0.009	0.022	0.944	0.905–0.986
F	Patellar tendon angle at 0–20 ° knee joint	63	0.013	0.045	0.894	0.818–0.976

(F: Females; M:males; SE: Standard Error)

### ROC curve analysis

**Fig. 3:**
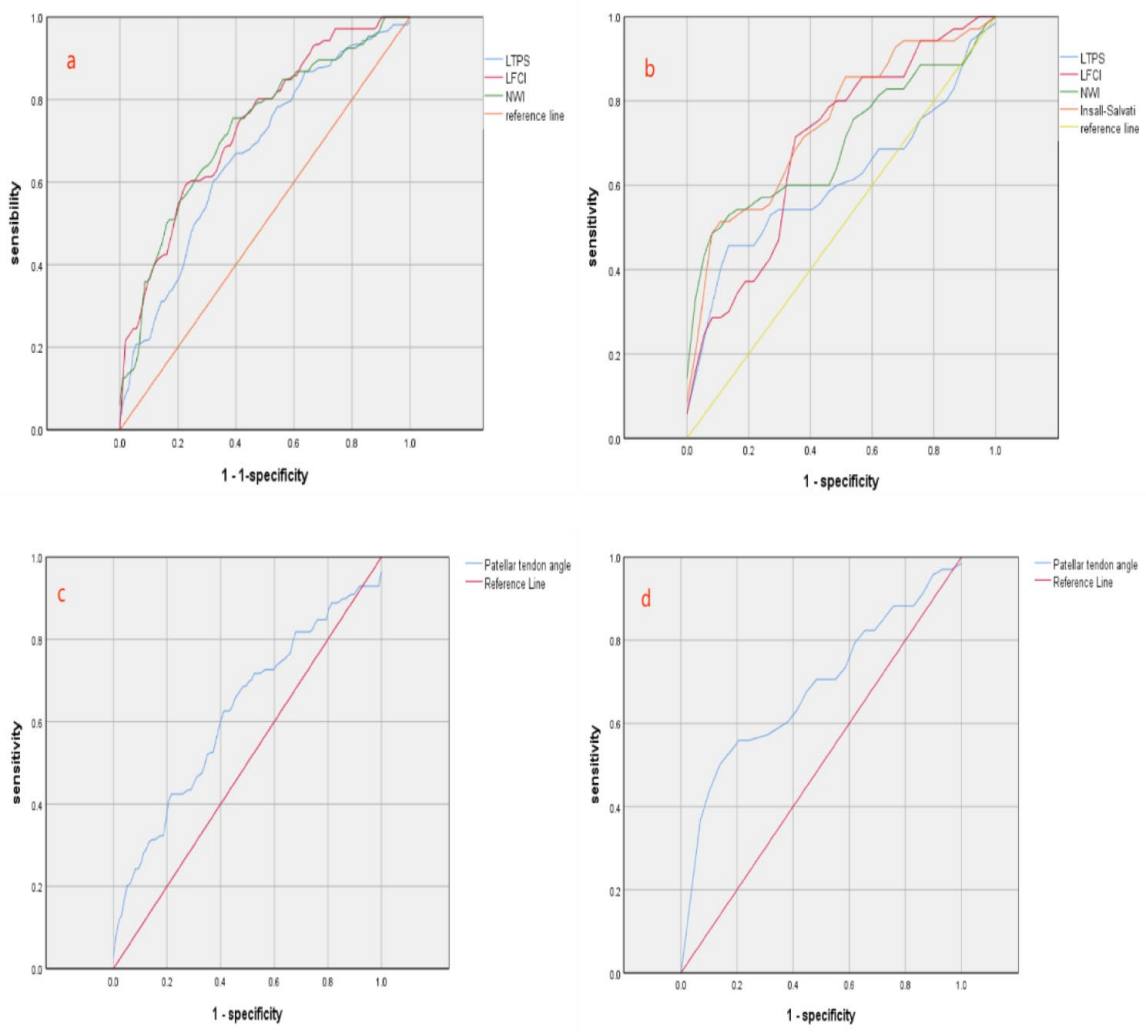
**a**: ROC curves for males LTPS, LFCI, and NWI. **b**: ROC curves for females LTPS, LFCI, NWI, and patellar index. **c**: ROC curve for males patellar tendon angle; **d**: ROC curve for females knee patellar tendon angle

ROC curve analysis for LPTS showed a higher cutoff for females (9.24) than males (6.22), with the males ROC area slightly exceeding the female’s. The LFCI cutoffs were close, at 0.567 for males and 0.551 for females, with the males curve area at 0.737, surpassing the female’s 0.696(Fig. 3).

The NWI’s ROC area was 0.728 for males and 0.700 for females, indicating high sensitivity and specificity, with females having a lower cutoff (0.268) than male(0.283).

The patellar index was significant for females, showing a large ROC area of 0.747 and a cutoff value of 1.218 (Table 3).

With a knee flexion angle < 20°, the patellar tendon angle’s significance varied, having a larger area for females (0.685) than males(0.622). The cutoff was 34.840 for male and 33.095 for females, highlighting gender differences in measurements (Fig. 3).

**Table 3:** ROC curve results

		area	cutoff	sensitivity	specificity
LTPS	M	0.669	6.22	0.604	0.686
	F	0.610	9.24	0.457	0.892
LFCI	M	0.737	0.567	0.594	0.781
	F	0.696	0.551	0.714	0.676
NWI	M	0.728	0.283	0.755	0.619
	F	0.700	0.268	0.543	0.865
Insall-Salvati	F	0.747	1.218	0.629	0.703
Tendon Angle	M	0.622	34.840	0.626	0.598
	F	0.685	33.095	0.500	0.897

(F: Females; M:males)

## Discussion

The most important finding of this study was that the LTPS is an anatomical risk factor for non-contact ACL injuries across genders. The role of the LTPS as a risk factor for ACL injuries has been debated in prior research. Dare et al., in a case-control study, discovered that an increased tibial plateau slope is associated with a heightened risk of ACL injuries [17]. Beynnon et al. observed that an increased tibial plateau slope elevates the risk of non-contact ACL injuries in females, yet they found no such correlation in males [12]. However, research by Hashemi et al. showed that the impact of the tibial plateau slope on ACL injuries shows no gender differences [18]. Our study corroborates that the LTPS is a risk factor in both genders. It also found that the cutoff value for the LTPS in males (6.22°) is significantly lower than in females (9.24°). In addition, the OR value was 1.293 for males and 1.275 for females, with the latter slightly higher. The average tibial plateau slope was 6.094° for males and 6.428° for females, suggesting that males may be more susceptible to the influence of the lateral tibial plateau slope.Increased LTPS may lead to increased anterior translation of the tibia, thereby increasing the tension on the ACL and making it more prone to injury.

The study confirmed the NWI as a significant risk factor for ACL injuries in both genders. Research by Prior et al. indicates that a smaller α angle and NWI are linked to increased ACL injury risks [7, 19, 20]. A narrower intercondylar notch, reflected by a smaller α angle, may cause ACL impingement, elevating injury chances [21]. Nonetheless, using visual inspection to classify the intercondylar notch shape could introduce inaccuracies. In Volkan Kızılgöz et al.‘s study on the impact of the intercondylar notch index on ACL injuries, both males and females were categorized, and statistically significant findings were observed. The cutoff value for males was 0.26, while for females, it was 0.27 [22]. Our study results indicate a significant association between NWI and ACL injuries, with OR of 0.767 for males and 0.807 for females. Additionally, the cutoff values were 0.283 for males and 0.268 for females. These findings suggest that the influence of NWI is more pronounced in males, as indicated by both the OR and cutoff values.

Another finding of this study is the association between the LFCI, which reflects the sphericity of the lateral femoral condyle [23], and ACL injuries. An increased LFCI indicates a deeper posterior condyle, associated with knee rotational instability, thus leading to ACL injuries [24]. The predictive value of the lateral femoral condyle morphology for ACL injuries remains a subject of debate. Hodel et al., in 2019, identified the morphology of the lateral condyle as one of the risk factors for ACL injuries [16]. However, Emma K et al.‘s study in 2022, analyzing both genders together and separately, did not find LFCI to be an independent predictor of ACL injury risk [8]. Neunghan Jeon et al.‘s 2022 study reiterated the increase in LFCI as a risk factor for ACL injury in females [9], but the measurements were based on knee X-rays rather than MRI, significantly influenced by the patient’s positioning. Our study identified LFCI as a common risk factor for ACL injuries, with cutoff values of 0.567 and 0.551 for males and females, respectively. The OR for females was 1.244, whereas for males, it was 1.147, indicating that the impact of LFCI on ACL injuries is more pronounced in females.

This study is the first to identify the patellar tendon angle at near knee extension (< 20°) as a predictor for ACL injuries. The ROC curve area was 0.685 for females and 0.622 for males. Additionally, the cutoff value for the patellar tendon angle was 33.095 for females and 34.840 for males. Moreover, the OR value for females was lower than for males (0.894 vs. 0.944), suggesting that changes in the patellar tendon angle have a greater protective effect against ACL injuries in females. The impact of the patellar tendon angle on ACL injuries might be due to the maximum shearing force exerted on the ACL when the knee is near extension [11], which decreases as the knee flexes further, reducing the anterior translation of the tibia caused by quadriceps contraction. Furthermore, the study identified the Insall-Salvati index as a risk factor for ACL injuries in females. Females have higher incidence of patella alta and patellar instability than males, which could highlight the significance of the patellar index in females ACL injuries [25]. This study also found that the average value of the Insall-Salvati index in females was significantly higher than in males. A. J. Degnan et al.’ study on the association between ACL injuries and patella alta in children aged 8–18 showed that ACL tears are associated with increased Insall-Salvati values [26]. Compared to those with normal patellar positioning, individuals with patella alta require greater knee flexion for patellar contact with the trochlea, creating contact stress between the femoral condyles and the patella. In certain situations, such as knee torsion, eversion, inversion, flexion, and extension, to prevent additional contact stress between the femoral condyles and the patella, a relative brief translation between the femur and tibia will increase. This translational change may subject the ACL to greater stress, thus increasing the risk of tears [27].

### Clinical relevance

This study conducted a comprehensive analysis separately for males and females, identifying various anatomical risk factors related to ACL injuries with certain gender differences and distinct cutoff values. Notably, this study is the first to find that the patellar tendon angle is related to ACL injuries in both male and female patients. Furthermore, the Insall-Salvati index has been identified as a risk factor for ACL injuries in females. These findings can contribute to targeted screening and preventative strategies for athletes, as well as aid in preventing reinjury after ACL reconstruction.

### Limitations

The study’s limitations include its single-institution subject pool, potentially limiting broader applicability. Despite using age and gender-matched controls, other variables like body metrics and activity levels were not controlled. The retrospective nature also introduces potential bias. Selection bias might be present due to the lack of clear explanation of patient inclusion and exclusion criteria, as well as the use of MRI in the control group. However, the study aimed to include consecutive patients meeting the criteria, and the use of MRI in the control group was necessary to ensure accurate measurements of anatomical factors. The matching process was based on age and gender, which are two critical factors influencing ACL injury risk, but a propensity score matching analysis was not performed due to the limited sample size. While suggesting clinical relevance for the patellar tendon angle in near-extension, a dynamic analysis might offer deeper insights. Additionally, the sample size was relatively limited, and a power analysis was not performed during the study design phase, which may affect the interpretation of the results. Despite these limitations, the study provides valuable insights into the gender-specific anatomical risk factors for ACL injuries, and the findings can serve as a foundation for future research and clinical applications.

## Conclusion

This study identifies the lateral tibial plateau slope, notch width index, lateral femoral condyle index, and patellar tendon angle at near-extension as risk factors for ACL injuries in both genders, with the Insall-Salvati index also implicated in females. These findings emphasize the complex role of common anatomical features in gender-specific ACL injury risk.

## Data Availability

The data were available from corresponding author upon reasonable request.

## Abbreviations

ACL: Anterior cruciate ligament
TPS: Tibial plateau slope
LTPS: Lateral tibial plateau slope
MTPS: Medial tibial plateau slope
MRI: Magnetic Resonance Imaging
LCFCR: Lateral condylar flexion circle radius
LCECR: Lateral condylar extension circle radius
LFCI: Lateral femoral condyle index
NWI: Notch width index
ROC: Receiver operating characteristic
